# BMP7 Gene Transfer via Gold Nanoparticles into Stroma Inhibits Corneal Fibrosis *In Vivo*

**DOI:** 10.1371/journal.pone.0066434

**Published:** 2013-06-14

**Authors:** Ashish Tandon, Ajay Sharma, Jason T. Rodier, Alexander M. Klibanov, Frank G. Rieger, Rajiv R. Mohan

**Affiliations:** 1 Harry S. Truman Memorial Veterans' Hospital, Columbia, Missouri, United States of America; 2 Mason Eye Institute, School of Medicine, University of Missouri, Columbia, Missouri, United States of America; 3 Departments of Chemistry and Biological Engineering, Massachusetts Institute of Technology, Cambridge, Massachusetts, United States of America; 4 College of Veterinary Medicine, University of Missouri, Columbia, Missouri, United States of America; University of Reading, United Kingdom

## Abstract

This study examined the effects of BMP7 gene transfer on corneal wound healing and fibrosis inhibition *in vivo* using a rabbit model. Corneal haze in rabbits was produced with the excimer laser performing -9 diopters photorefractive keratectomy. BMP7 gene was introduced into rabbit keratocytes by polyethylimine-conjugated gold nanoparticles (PEI2-GNPs) transfection solution single 5-minute topical application on the eye. Corneal haze and ocular health in live animals was gauged with stereo- and slit-lamp biomicroscopy. The levels of fibrosis [α-smooth muscle actin (αSMA), F-actin and fibronectin], immune reaction (CD11b and F4/80), keratocyte apoptosis (TUNEL), calcification (alizarin red, vonKossa and osteocalcin), and delivered-BMP7 gene expression in corneal tissues were quantified with immunofluorescence, western blotting and/or real-time PCR. Human corneal fibroblasts (HCF) and *in vitro* experiments were used to characterize the molecular mechanism mediating BMP7’s anti-fibrosis effects. PEI2-GNPs showed substantial BMP7 gene delivery into rabbit keratocytes in vivo (2×10^4^ gene copies/ug DNA). Localized BMP7 gene therapy showed a significant corneal haze decrease (1.68±0.31 compared to 3.2±0.43 in control corneas; p<0.05) in Fantes grading scale. Immunostaining and immunoblot analyses detected significantly reduced levels of αSMA (46±5% p<0.001) and fibronectin proteins (48±5% p<0.01). TUNEL, CD11b, and F4/80 assays revealed that BMP7 gene therapy is nonimmunogenic and nontoxic for the cornea. Furthermore, alizarin red, vonKossa and osteocalcin analyses revealed that localized PEI2-GNP-mediated BMP7 gene transfer in rabbit cornea does not cause calcification or osteoblast recruitment. Immunofluorescence of BMP7-transefected HCFs showed significantly increased pSmad-1/5/8 nuclear localization (>88%; p<0.0001), and immunoblotting of BMP7-transefected HCFs grown in the presence of TGFβ demonstrated significantly enhanced pSmad-1/5/8 (95%; p<0.001) and Smad6 (53%, p<0.001), and decreased αSMA (78%; p<0.001) protein levels. These results suggest that localized BMP7 gene delivery in rabbit cornea modulates wound healing and inhibits fibrosis *in vivo* by counter balancing TGFβ1-mediated profibrotic Smad signaling.

## Introduction

Trauma, infection, or chemical/surgical injury to the cornea can cause fibrosis/scarring resulting in loss of corneal transparency. Though corneal scarring is the third leading cause of blindness worldwide and affects over one million Americans every year [1], no effective therapy is yet available to treat corneal scarring. Steroids have been used, but their effectiveness remains controversial and they have significant side effects [2], [3]. Mitomycin C is commonly used in clinic to treat laser surgery-induced corneal scarring [4]–[6]. However, its use has been associated with serious side effects both in preclinical and clinical studies [7]–[11]. Due to the lack of effective and safe drugs, many cases of corneal scarring require corneal transplantation. Despite its high success rate, corneal transplantation poses the challenges of postsurgical complications and the limited availability of high quality donor corneas. Thus, there is a need for newer and effective treatments for corneal scarring.

Innovations in nanotechnology have generated nanoparticles that can potentially be used as gene delivery vectors, largely due to their ability to carry therapeutic molecules with high efficiency and low toxicity into targeted cells/tissues. A variety of nanoparticles recently have been tested for their potential as a gene therapy vectors for various cell types [12]–[15]. Some of these nanoparticles such as gold, albumin, 1,2-dioleoyl-3-trimethylammonium-propane (DOTAP), 1,2-dioleoyl-sn-glycero-3-phosphoethanolamine (DOPE), and poly(lactic-co-glycolic acid) (PLGA) have been tested for gene therapy to treat corneal diseases including fibrosis or neovascularization [16]–[19]. Recently, we identified that polyethylenimine-conjugated gold nanoparticles (PEI2-GNPs) are capable of delivering genes into corneal cells both *in vitro* and *in vivo* with high potency and low-moderate toxicity [20]. The PEI2-GNPs are a promising non-viral vector for ocular gene therapy due to their many unique features such as ease of synthesis, high plasmid binding capacity, biocompatibility and low immunogenicity. These characteristics and high transfection efficiency prompted us to hypothesize that PEI2-GNPs-mediated targeted gene delivery into keratocytes of rabbit stroma can provide required levels of therapeutic genes to treat corneal diseases.

Bone morphogenetic proteins (BMP) are a large family of growth factors with more than 10 members [21], [22]. The cornea has been reported to express several BMPs and their receptors [23]–[27]. We have previously reported that BMP2 and BMP4 modulate keratocyte proliferation and apoptosis in the human cornea [23]. Among other BMP family members, BMP7 has been shown to play a pivotal role in eye development during embryogenesis, and BMP7- knockout mice have anophthalmia [28], [29]. In adult animals, endogenous BMP7 levels in the eye and other organs decline but exogenous BMP7 administration has been demonstrated to attenuate fibrosis [30], [31]. In the cornea, therapeutic effects of BMP7 have been examined by topical application of recombinant protein or adenovirus-mediated gene delivery in a mouse model of chemical injury [32], [33]. Although a significant beneficial effect was noted on epithelial regeneration, the effects on restoration of corneal transparency were less remarkable [32], [33]. Rapid clearance and the possibility of adenoviral vector-induced inflammatory response may have undermined the beneficial effects of BMP7 in these studies and necessitate testing of additional gene delivery vectors. Furthermore, beneficial effects of BMP7 need to be examined in a TGFβ-driven model free from such confounding effects as neovascularization and inflammation observed in chemical injury models. The purpose of the present study was to evaluate the effect of PEI2-GNPs-mediated BMP7 gene therapy on corneal fibrosis using an *in vivo* animal model of laser ablation-induced corneal fibrosis.

## Materials and Methods

### *In vivo* Rabbit Model of Corneal Fibrosis

Eighteen female New Zealand White rabbits (Harlan laboratories, Indianapolis IN) weighing 2.0 to 3.0 kg each were used in this study. The Institutional Animal Care and Use Committee of the University of Missouri-Columbia and Harry S. Truman Memorial Veterans' Hospital approved the study. All animals were treated in accordance with the ARVO Statement for the Use of Animals in Ophthalmic and Vision Research. A well-established excimer laser based rabbit corneal fibrosis model was used [34]. Rabbits were anesthetized by intramuscular injection of a mixture of ketamine hydrochloride (50 mg/kg) and xylazine hydrochloride (10 mg/kg) and 2 to 3 drops of 0.5% proparacaine hydrochloride (Alcon, Fort Worth, TX) solution were instilled on the cornea for local anesthesia. The corneal epithelium was removed by gentle scraping with a #64 Beaver blade (BD Biosciences, Franklin Lakes, NJ), and -9 diopter photorefractive keratectomy (PRK) was performed creating a 6-mm ablation zone with the excimer laser (Alcon). This PRK technique has been shown to consistently produce fibrosis and myofibroblasts in the rabbit cornea that peaks at 4 weeks [35], [36].

### Nanoparticle Transfection

PEI2-GNPs were synthesized by conjugation of thiol modified 2-kDa PEI to GNPs as described earlier [37]. The PEI2-GNPs DNA polyplexes were prepared at a nitrogen-to- phosphate (N-P) ratio of 180 by mixing 37.5 µl of 150 mM PEI2-GNPs with 10 µg of plasmid DNA (pTRUF11 expressing GFP gene or BMP7 under control of CMV+chicken beta actin promoter) and diluting with buffered salt solution (BSS) containing 10% glucose (w/v). The resulting solution was incubated at 37°C for 30 minutes prior to use.

Rabbits were divided into three groups. Only one eye of each rabbit was used for experiment. The animals Group-I received BSS, Group-II received PEI2-GNP-GFP or naked plasmid transfection solution and Group-III received PEI2-GNP-BMP7 plasmid transfection solution for 5 minutes topically immediately after PRK using a cloning cylinder [38]. The cloning cylinder increases vector contact to the cornea and restricts gene transfer in the neighboring ocular cells and limits toxicity. The contralateral eyes served as naïve controls.

### Slit Lamp Biomicroscopy

The level of corneal haze in live rabbits was gauged by slit lamp microscope (BX 900 Slit Lamp, Haag-Streit USA, Mason, OH) examinations before PRK and 4 weeks after PRK, as described earlier [34], [39]. Grade 0 was a completely clear cornea; grade 0.5 had trace haze seen with careful oblique illumination with the slit lamp biomicroscope; grade 1 was more prominent haze not interfering with the visibility of fine iris details; grade 2 was mild obscuration of iris details; grade 3 was moderate obscuration of the iris and lens; and grade 4 was complete opacification of the stroma in the area of ablation. Haze grading was performed by two observers (RRM, FGR, AS, AT and/or JTR) in a masked manner.

### Euthanasia and Tissue Collection

Four weeks after PRK and vector application, the rabbits were humanely euthanized with a pentobarbitone (150 mg/kg) overdose while under general anesthesia. Corneas of six rabbits of each group were removed with forceps and sharp Westcott scissors, embedded in liquid optimal cutting temperature (OCT) compound (Sakura FineTek, Torrance, CA) in a 24×24×5-mm mold (Fisher Scientific, Pittsburgh, PA), and snap frozen. Frozen tissue blocks were maintained at −80°C for future use. Tissue sections were cut 7 µm thick with a cryostat (HM 525M; Microm GmbH, Walldorf, Germany), placed on 25×75×1-mm microscope slides (Superfrost Plus; Fisher Scientific), and maintained frozen at −80°C until staining.

### Immunofluorescence

Immunofluorescence staining for αSMA (myofibroblast marker) fibronectin (tissue remodeling marker) and osteoclacin (osteoblast marker) was performed by incubating corneal sections with 5% bovine serum albumin for 30 min at room temperature followed by mouse monoclonal primary αSMA antibody (1∶200 dilution, M0851; Dako, Carpinteria, CA), goat polyclonal primary fibronectin antibody (1∶200 dilution, sc6952; Santa Cruz Biotechnology, Santa Cruz, CA), or mouse monoclonal primary osteocalcin antibody (1∶100 dilution, sc376835; Santa Cruz Biotechnology, Santa Cruz, CA) for 90 min. For detection of the primary antibody, the sections were exposed to Alexa 488 goat anti-mouse IgG secondary antibody (1∶1000 dilution, A11001; Invitrogen Inc., Carlsbad, CA) or Alexa 594 donkey anti-goat IgG secondary antibody (1∶1000 dilution, A11058; Invitrogen) for 60 min. F-actin immunostaining was performed by incubating the tissue sections in Alexa 594-conjugated phalloidin (1∶50 dilution, A12381; Invitrogen) for 90 min. SMA+, Fibronectin+ and F-actin+ cells in six randomly selected non-overlapping full-thickness central corneal columns extending from the anterior stromal surface to the posterior stromal surface were counted. The diameter of each column was that of a 200 X or 400 X magnification field. NIH Image J software and/or manual cell counting were used for quantification.

The immunologic reaction to PEI2-GNPs-mediated BMP7 gene therapy was examined by performing CD11b (BDB550282; BD Pharmingen, San Jose, CA) and F4/80 (MCA497; Serotec, Raleigh, NC) immunostaining in rabbit corneal sections using rat anti-mouse antibody [10], [26]. Tissue sections were incubated at room temperature with the CD11b primary antibody at a 1∶50 dilution in a 1× HEPES buffer containing 5% BSA for 90 min, followed by goat anti-rat IgG secondary antibody (Alexa Fluor 594; Invitrogen) at a 1∶1000 dilution for 60 min. After completion of immunostaining, tissue sections were mounted in medium containing DAPI (Vectashield; Vector Laboratories, Burlingame, CA), viewed, and photographed under a fluorescence microscope (Leica, Deerfield, IL) equipped with a digital camera system (SpotCam RT KE; Diagnostic Instruments, Sterling, MI).

Negative controls for each immunocytochemistry experiment were irrelevant isotype-matched primary antibodies, omission of primary or secondary antibody, secondary antibody alone, and tissue sections of the naïve eye.

### TUNEL Assay

A TUNEL assay was performed according to the manufacturer's instructions in acetone-fixed rabbit corneal sections using a fluorescent detection assay (ApopTag; Millipore) that detects apoptosis and, to a lesser extent, necrosis. Rabbit tissue section of 4 hours mechanical corneal scrape was used as a positive control. Unwounded rabbit corneal tissue section was included as a negative control (data not shown).

### Histology

To detect any tissue calcium deposits, alizarin red S staining and von Kossa staining was performed on the rabbit corneal tissue sections. Horse hoof tissues were used as positive controls. For alizarin red S staining, tissue sections were exposed to 1% aqueous solution of alizarin red for 5 minutes followed by hematoxylin staining to detect nuclei. For vonKossa staining, tissues were treated with 5% silver nitrate followed by 20 minutes UV exposure, washing and 5 minutes treatment with 2% sodium thiosulphate. Nuclei were stained with nuclear fast red.

### Quantification of Gene Copy Number

The copies of delivered BMP7 plasmid were determined using real-time PCR. DNA was isolated from the post-mortem rabbit corneas using DNeasy kit (Quiagen). The standard curve was plotted using a 10 fold serial dilution of copies of BMP7 pTRUF11 plasmid. Forward primer TGG AGA CGC TGG ATGG and reverse primer CTG CGG AAG TGG ACCT were used for running real-time PCR with the following settings: 95°C for 10 min, 40 cycles at 95°C for 15 s, and 60°C for 60 s.

### Human Corneal Fibroblasts and Transfection

Primary human corneal fibroblast (HCF) cultures were generated from donor human corneas obtained from the Heartland Eye Bank, St. Louis, Missouri following earlier reported method [34]. To generate myofibroblast cultures, HCFs were seeded using DMEM containing 10% serum, after 8–12 h switched to serum-free medium containing TGFβ1 (5 ng/ml). The myofibroblast cultures were fed with fresh serum-free TGFβ1 containing medium every 24 h.

Transfections of HCF cultures were performed by incubating the cultures with 200 µl of PEI2-GNPs-BMP7 transfection solution in 2 ml DMEM medium containing 10% serum for 6 hours. Thereafter, cultures were washed with PBS and allowed to grow in serum-free conditions in the presence of TGFβ1 (5 ng/ml) for 30–36 hours.

### Western Blotting

Cell lysates were prepared in RIPA protein lysis buffer containing protease inhibitor cocktail (Roche Applied Sciences, Indianapolis, IN) and phosphatase inhibitor. Protein samples were prepared for electrophoresis by heating at 90°C for 10 min. The samples were transferred onto polyvinylidene difluoride (PVDF) membranes (iBlot apparatus; Invitrogen), and the proteins were detected with primary antibodies for the following: BMP7 (1∶100 dilution, MAB3541; R&D systems, Minneapolis, MN) αSMA (1∶200 dilution, M0851; Dako, Carpinteria, CA), phospho-Smad1/5/8 (1∶100 dilution; Cell signaling), Smad6 (1∶100 dilution; Abcam) and β-actin (1∶100 dilution; Santa Cruz) followed by alkaline phosphatase–conjugated anti-rabbit secondary antibody (Fisher Scientific). After the membrane was washed three times in 0.05% Tween-20 in Tris-buffered saline (pH 8.0) for 5 min each, the blot was developed using the nitroblue tetrazolium (NBT)/5-bromo-4-chloro-3-indolylphosphate (BCIP) method.

### Statistical Analyses

The results of corneal haze grading and SMA quantification were expressed as the mean ± SEM. Statistical analysis was performed using the Student's t-test or the Wilcoxon rank sum test, p<0.05 indicated significance.

## Results

### PEI2-GNPs Gene Transfer in Rabbit Cornea *in vivo*

**Figure 1:**
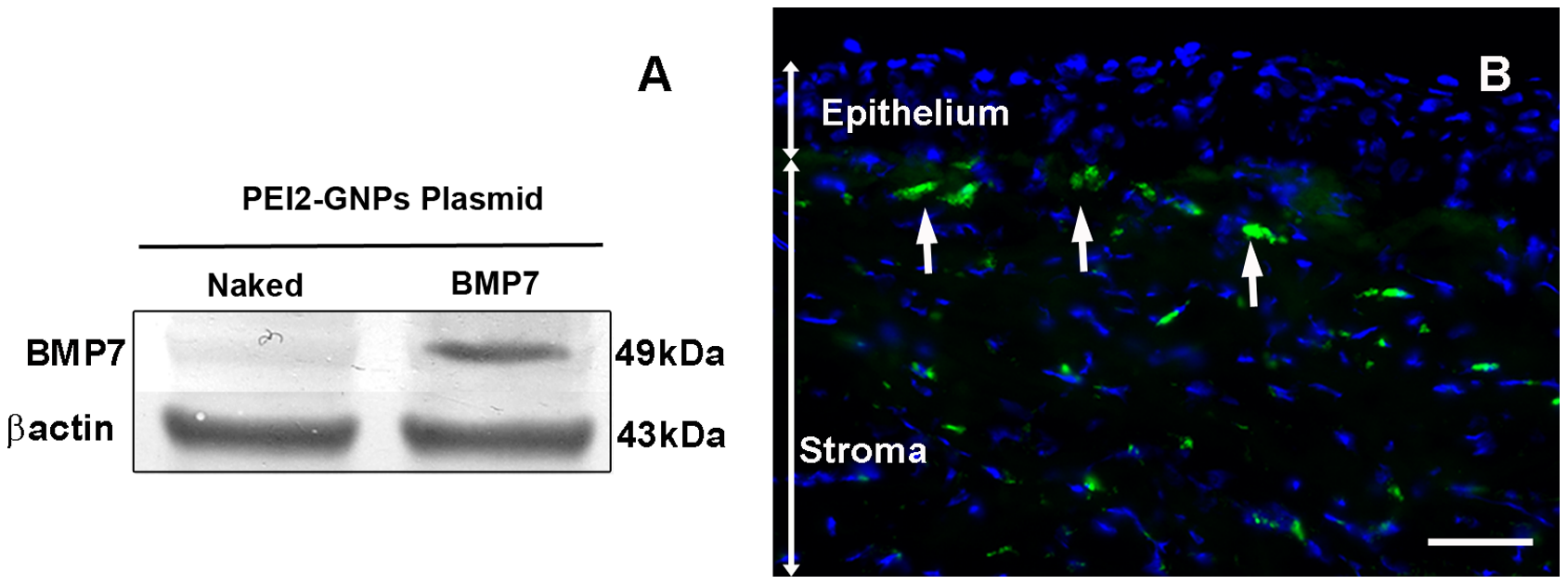
Western blot (A) and fluorescence microscopy image (B) showing delivered BMP7 protein expression and localization of delivered transgene in rabbit corneas collected 4 weeks after a single 5 min topical application transfection solution made of PEI2-GNPs and plasmid expressing BMP7 (A) or GFP gene (B). Scale bar denotes 100 µm.

The *in vivo* corneal gene transfer ability of PEI2-GNPs-BMP7 transfection solution was examined by measuring delivered-BMP7 protein levels and gene copies using western blotting and real-time PCR, respectively. As shown in Figure 1A, PEI2-GNPs-BMP7 exposed rabbit corneal tissue lystaes showed substantial level of BMP7, thus confirming a successful BMP7 gene delivery and protein expression (Figure 1A). For detecting the localization of PEI2-GNPs-mediated gene transfer, GFP immunofluorescence was examined in the rabbit corneal tissue sections. Figure 1B showed substantial GFP expression in the anterior and mid stroma of the rabbit corneas after a single topical application of PEI2-GNPs-GFP plasmid transfection mixture, thus confirming targeted gene delivery into rabbit keratocytes *in vivo* with PEI2-GNPs.

**Figure 2:**
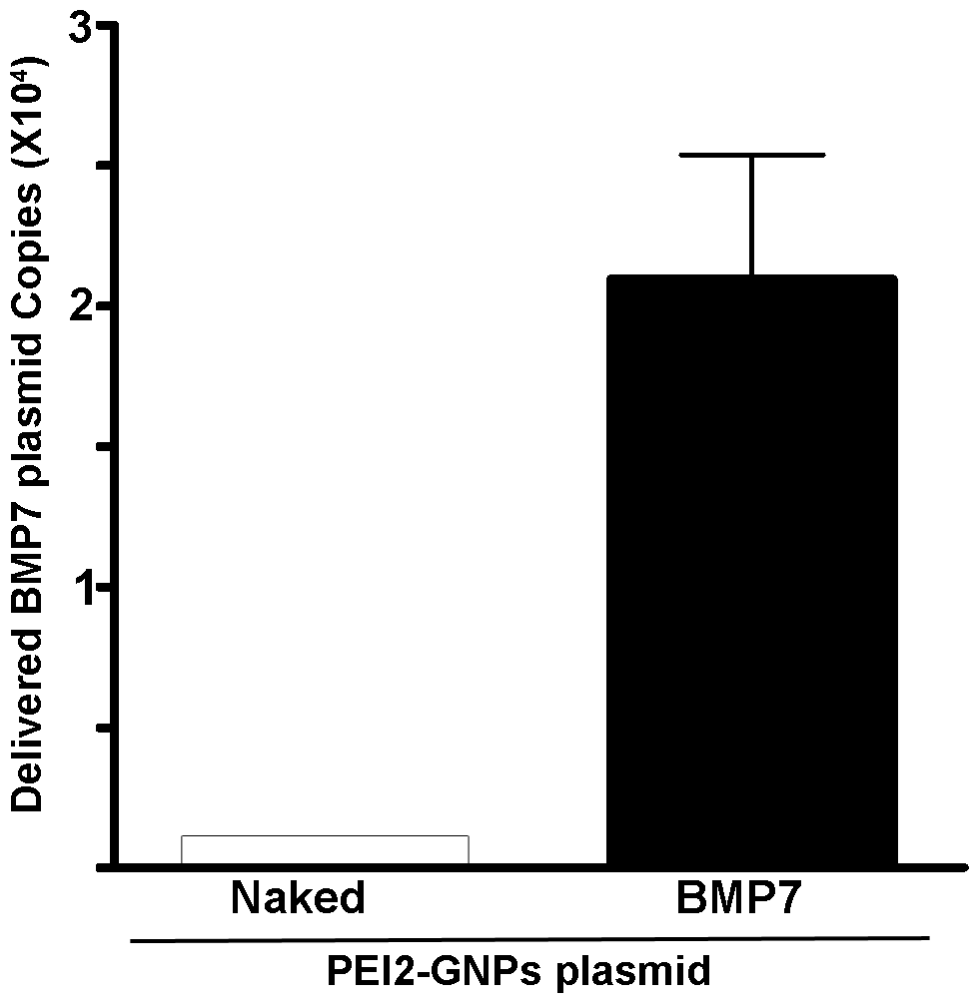
Real-time PCR data showing quantification of delivered BMP7 gene copies in rabbit corneas after a single 5 min topical application of PEI2-GNPs-BMP7 transfection solution.

The BMP7 gene copies delivered into rabbit cornea with PEI2-GNPs were quantified with real-time PCR and are shown in Figure 2. Detection of 2×10^4^ BMP7 gene copies per µg of DNA demonstrated PEI2-GNPs are efficient vector for delivering genes into rabbit corneas *in vivo* (Figure 2).

### BMP7 Gene Transfer Inhibits Corneal Fibrosis *in vivo*

**Figure 3:**
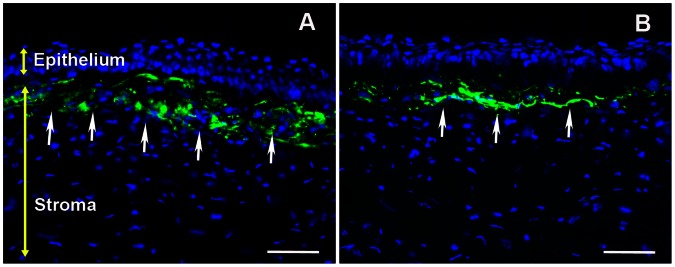
Representative images showing immunofluorescence staining for αSMA (green), a myofibroblast marker, in the stroma of laser-ablated corneal tissue sections obtained from rabbit eyes that received a single 5 min topical application of transfection solution; PEI2-GNPs naked plasmid (A) or BMP7 expressing plasmid (B). Scale bar denotes 100 µm.

The presence of myofibroblasts is a characteristic feature of laser-induced corneal haze. Typically, these myofibroblasts express bundles of filamentous proteins and therefore can be readily detected by staining for αSMA or F-actin. As expected, corneal tissue sections obtained from the rabbits 4 weeks after PRK showed substantial αSMA+ cells beneath corneal epithelium in the anterior stroma, thus confirming myofibroblast formation (Figure 3A). BMP7-trasnfected rabbit corneal tissue sections showed remarkably fewer αSMA+ cells compared to laser-ablated un-transfected corneas suggesting that BMP7 gene therapy attenuated laser-induced myofibroblast formation (Figure 3B).

**Figure 4:**
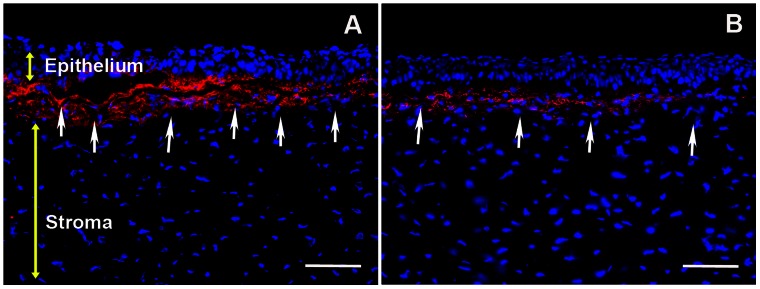
Representative images showing immunofluorescence staining for fibronectin (red) in the stroma of laser ablated corneal tissue sections obtained from rabbit eyes that received a single 5 min topical application of transfection solution made of PEI2-GNPs naked plasmid (A) or BMP7 expressing plasmid (B). Scale bar denotes 100 µm.

Increased fibronectin levels are reported during *in vivo* corneal wound healing, and fibronectin matrix assembly has been shown to facilitate TGFβ-mediated myofibroblast induction *in vitro* [34, 39, and 40]. Figure 4A shows high fibronectin staining in the anterior stroma of PRK treated rabbit corneal tissue sections. PEI2-GNPs-BMP7 gene therapy caused a notable decrease in PRK-induced fibronectin levels (Figure 4B).

**Figure 5:**
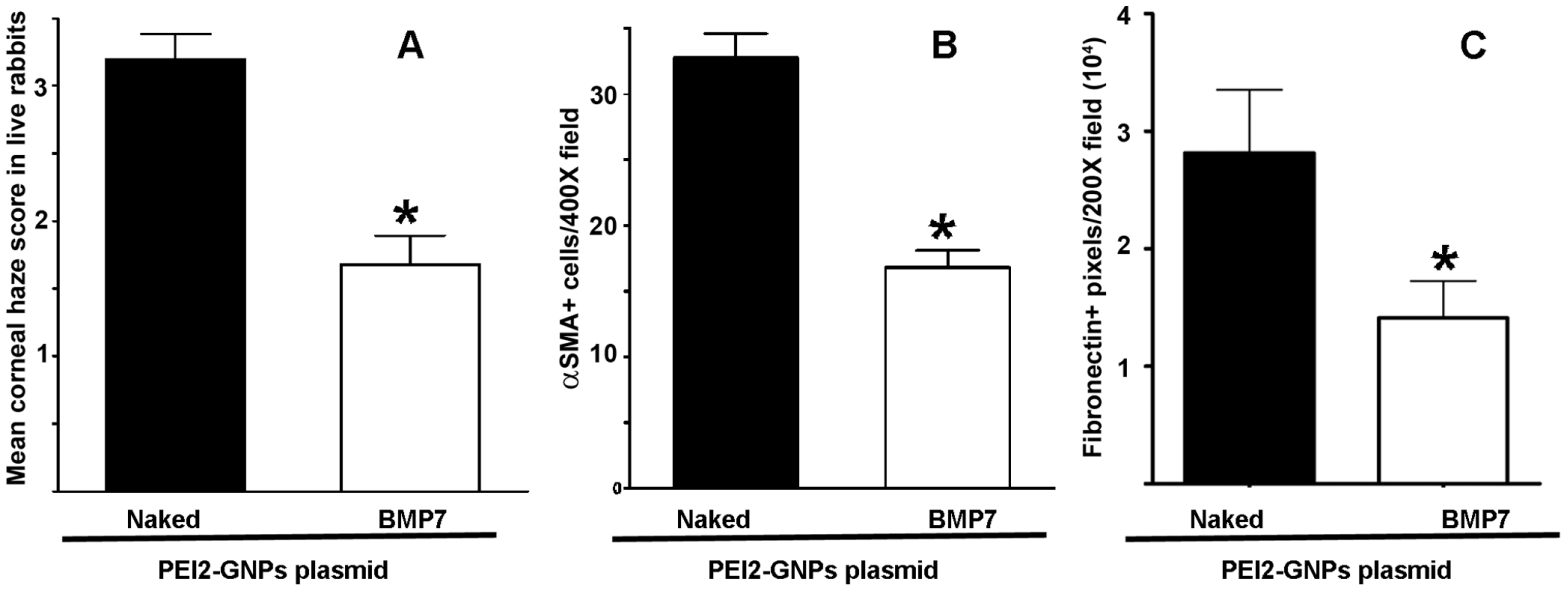
Quantification of corneal haze in live animals (A), and SMA+ (B) and fibronectin+ (C) cells in corneal tissues 4-week after laser ablation and +/− BMP7 gene transfer. PEI2-GNPs mediated BMP7 gene transfer significantly reduced (p<0.05) in corneal haze, and decreased SMA+ cells (p<0.001) and fibronectin-immunostained area (p<0.01).

Figure 5 shows quantification of corneal haze in live rabbits using Fantes scale (Panel A) and αSMA+ (Panel B) and fibronectin+ (Panel C) cells in un-transfected and PEI2-GNPs-BMP7 transfected laser-ablated rabbit corneal tissues. Corneas that were subjected to laser ablation but received no transfection solution showed a strong fibrotic response 4 week after PRK as evident from the haze score of 3.2±0.43 (Figure 5A) whereas BMP7 gene transfer showed a significant (p<0.05) attenuation of laser-induced corneal haze as noted by low haze score of 1.68±0.31 (Figure 5A). The changes noted in live animal eyes were also validated by the histological findings. The PEI2-GNPs-mediated BMP7 gene delivery caused a significant 46±5% (p<0.001) decrease in αSMA+ cells as compared to corneas that received no gene delivery (Figure 5B). Quantification of fibronectin stained area in BMP7-delivered corneas detected a significant 48±5% (p<0.01) reduction in fibronectin (Figure 5C).

### Effects of Localized BMP7 Gene Transfer on Keratocyte Apoptosis, Immune Reaction and Calcification in Rabbit Corneas *in vivo*

**Figure 6:**
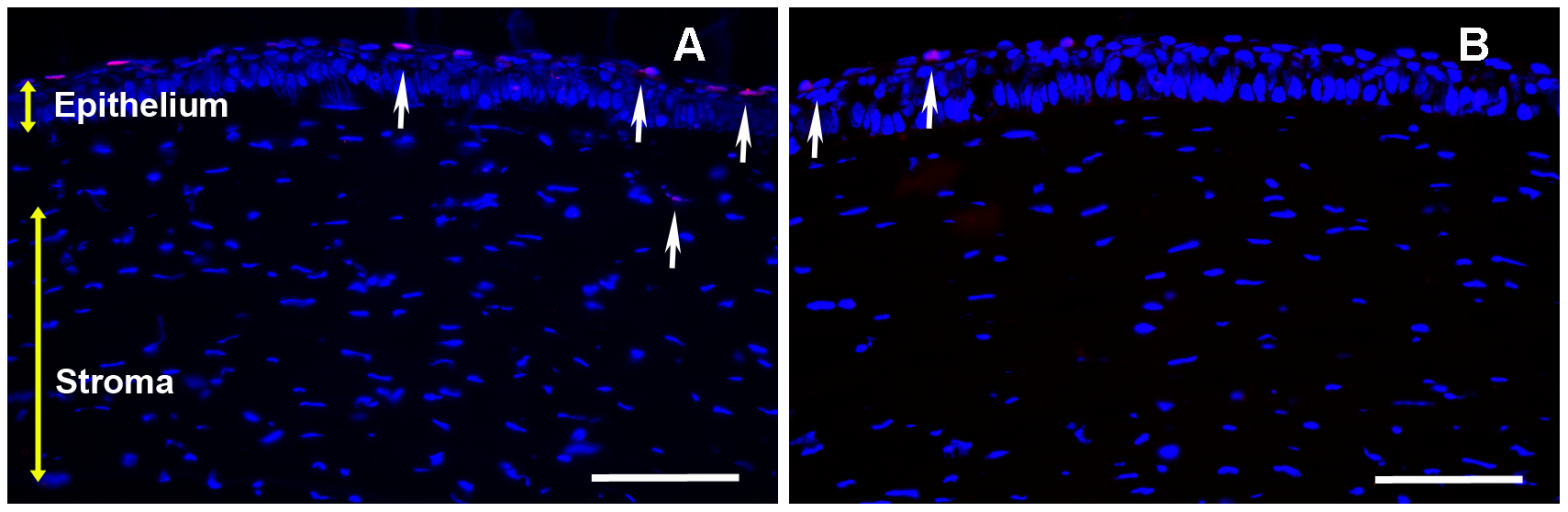
Representative images showing TUNEL assay data of rabbit corneas collected 4 weeks after laser ablation and one 5 min topical application of PEI2-GNPs naked plasmid (A) or BMP7 plasmid (B) transfection solution. Tissue sections of rabbit corneas transfected with naked plasmid (A) or BMP7 plasmid (B) depicted no significant difference in TUNEL+ cells between the two groups. As expected many TUNEL+ cells in corneal epithelium were observed due to normal epithelium turnover. Scale bar denotes 100 µm.

The effect of PEI2-GNPs-mediated BMP7 gene transfer on keratocyte apoptosis was determined by counting TUNEL+ cells in the stroma. As evident from Figure 6, few TUNEL+ cells (2–8 apoptotic cells) were detected in the stroma of laser-ablated un-transfected (Figure 6A) and BMP7-trasfected (Figure 6B) rabbit corneas. The quantification of TUNEL+ cells revealed no significant difference between these two groups. Corneal epithelium of both, un-transfected and BMP7-transfected corneas showed several TUNEL+ cells that represent normal replenishment of corneal epithelium via apoptosis.

**Figure 7:**
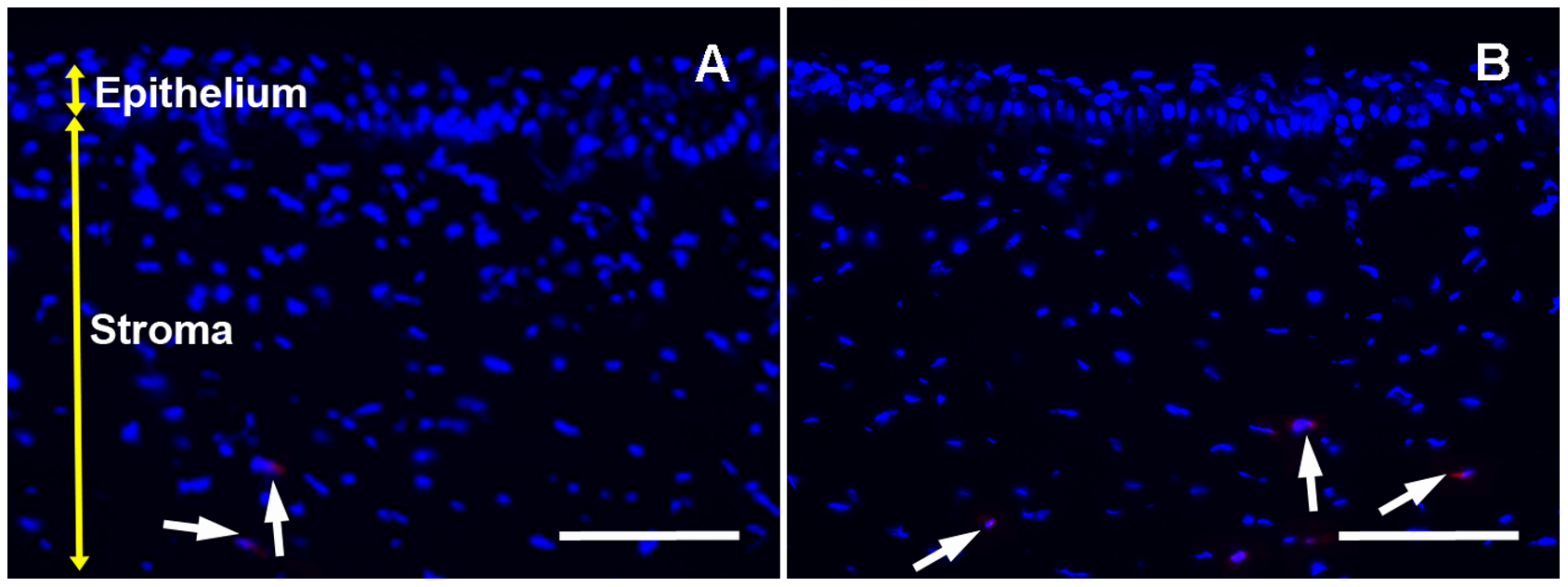
Representative images showing CD11b immunostaining in rabbit corneas collected 4 weeks after laser ablation without (A) and with BMP7 (B) gene transfer. No significant difference in the CD11b+ cells in tissue sections of rabbit corneas transfected with PEI2-GNPs naked (A) or BMP7 (B) plasmid was detected. Scale bar denotes 100 µm.

Figure 7 shows the results of CD11b immunostaining in the tissue sections obtained from untreated and PEI2-GNPs-BMP7- treated rabbit corneas. Occasional CD11b+ cells were detected in untreated (Figure 7A) and PEI2-GNPs-BMP7 treated rabbit corneas (Figure 7B). Similar results were found with F4/80 immunohistochemistry (data not shown). The quantification revealed no statistically significant difference in CD11b+ or F4/80+ cells between the 2 groups (un-transfected and BMP7-transfected corneas).

**Figure 8:**
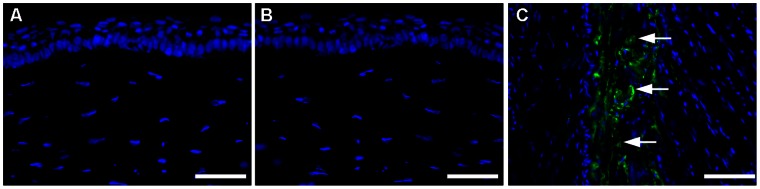
Representative osteocalcin immunofluorescence of rabbit corneas collected 4 weeks after laser ablation and PEI2-GNPs naked (A) or BMP7-expressing plasmid (B) transfection solution treatment, and tissue sections of horse hoof with laminitis (C). Neither un-transfected nor BMP7-transfected rabbit corneas showed osteocalcin+ cells. This suggests that localized BMP7 overexpression in the cornea does not cause osteoblast recruitment. The horse hoof of laminitis, positive control, showed many osteocalcin+ cells (C). Scale bar denotes 100 µm.

**Figure 9:**
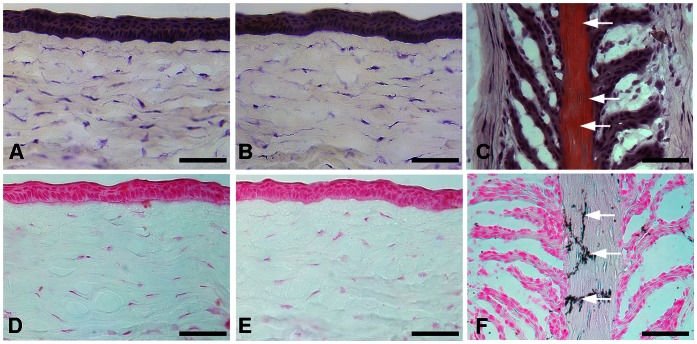
Representative alizarin red (A-C) and vonKossa (D-F) staining in rabbit corneas and horse hoof laminitis tissue sections. Rabbit corneas collected 4 weeks after laser ablation and PEI2-GNPs naked (A, D) or BMP7-expressing plasmid (B, E) treatment showed no alizarin red (A, B) or vonKossa (D, E) staining. This suggests that localized BMP7 gene transfer in rabbit cornea does not cause calcium deposits. Positive controls of horse hoof laminitis tissues (C, F) showed strong alizarin red (C) and vonKossa (F) staining. Scale bar denotes 100 µm.

The biological functions of BMP7 are tissue specific. In bone tissue BMP7 has been reported to cause osteoblast differentiation and calcification [41]. Conversely, BMP7 is shown to prevent calcification in the vascular smooth muscle [42]. To rule out the possibility that BMP7 overexpression does not cause osteoblast recruitment or calcification in the cornea, we performed osteocalcin immunostaining specific for osteoblast (Figure 8), and alizarin red (Figure 9A–C). and vonKossa staining for detecting calcium deposits (Figure 9D–F). PEI2-GNP-mediated localized BMP7 gene transfer in rabbit corneas did not show any osteocalcin+ cells (Figure 8B) or calcium deposits (Figure 9B and Figure 9E) like corneal sections of un-transfected rabbit corneas (Figure 8A, Figure 9A and Figure 9D). This data confirmed that localized BMP7 gene delivery in rabbit cornea does not cause osteoblast recruitment or calcification. The positive controls, tissue sections of horse hoof with laminitis, showed many osteocalcin+ cells (Figure 8C) and large calcium deposits (Figure 9C and Figure 9F).

### Effects of BMP7 Gene Transfer on Smad Pathway

**Figure 10:**
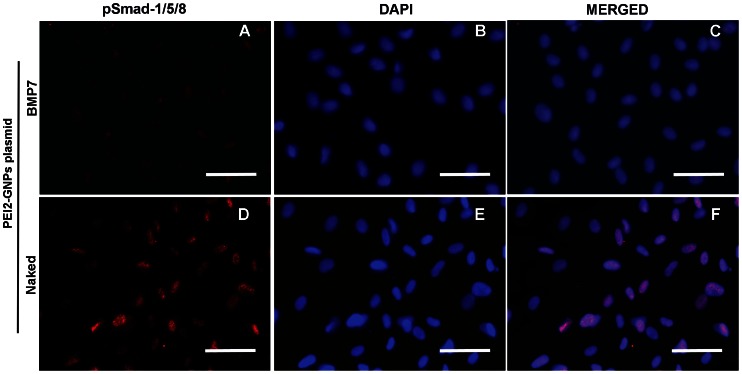
Representative immunofluorescence image showing pSmad-1/5/8 (Red) staining in HCFs transfected with PEI2-GNPs naked plasmid (A–C) or BMP7-expressing plasmid (D–F). BMP7 overexpressing HCFs showed significantly higher pSmad-1/5/8 nuclear localization (C; 88±5%; p<0.0001). Nuclei are stained blue. Scale bar denotes 100 µm.

**Figure 11:**
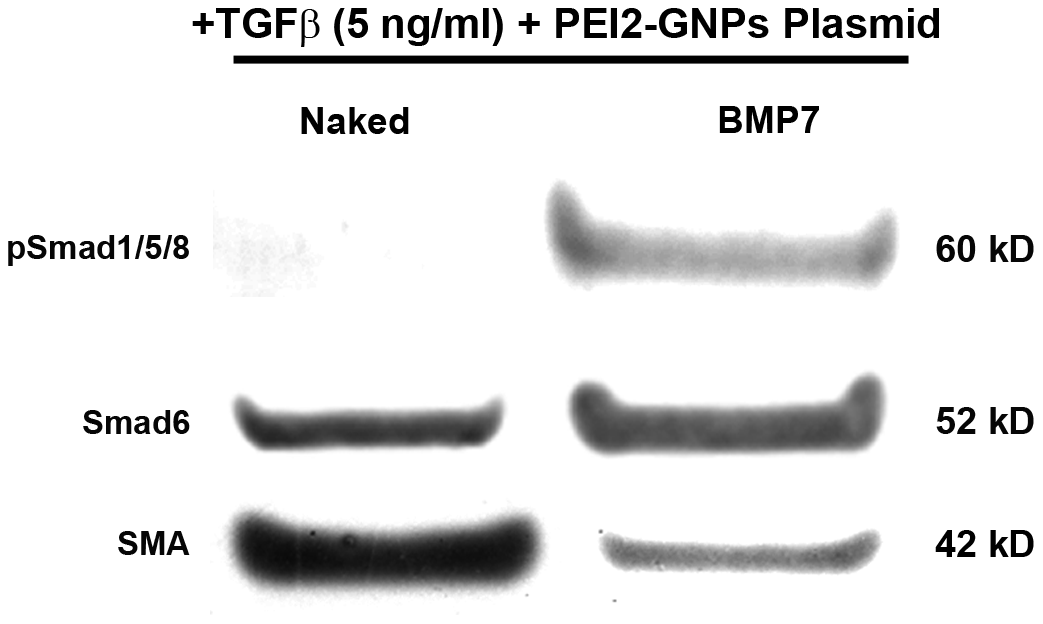
Representative western blotting demonstrating pSmad1/5/8, Smad6 and αSMA protein levels in HCFs transfected with PEI2-GNPs naked plasmid or BMP7-expressing plasmid. BMP7-transfected HCFs grown in the presence of TGFβ demonstrated a statistically significant increased pSmad-1/5/8 (95%; p<0.001) and Smad6 (53%, p<0.001), and decreased αSMA (78%; p<0.001) protein levels.

Reports on the expression of BMP7 and its receptors in the cornea are inconclusive. BMP7 is known to compete with TGFβ signaling. Phosphorylation of Smad1/5/8 is the first step involved in BMP7 signaling and its biological activity. In order to confirm that BMP7 affects anti-TGFβ fibrotic signaling in corneal fibroblasts we performed immunofluorescence and western blotting for pSmad1/5/8 in HCFs transfected with BMP7-expressing or naked plasmid with PEI2-GNPs and grown in the presence or absence of TGFβ1. Figure 10 shows the immunofluorescence staining for pSmad-1/5/8 in human corneal fibroblasts transfected with BMP7 or naked plasmid and grown in the absence of TGFβ1. The BMP7-transfected HCFs showed significantly increased 88±5% pSmad-1/5/8 nuclear localization (p<0.0001, Figure 10F) compared to naked plasmid transfected HCF cultures that showed 12±3% pSmad-1/5/8 nuclear immunostaining (Figure 10C). Figure 11 shows western blot quantification of pSmad-1/5/8, Smad6 and αSMA proteins expression in human corneal fibroblasts transfected with BMP7 or naked plasmid cultured in the presence of TGFβ1. BMP7-transfected HCFs grown in the presence of TGFβ1 demonstrated a statistically significant increased pSmad-1/5/8 (95%; p<0.001) and Smad6 (53%, p<0.001), and decreased αSMA (78%; p<0.001) protein levels compared to the samples of naked plasmid transfected HCFs grown under similar conditions (Figure 11). The detection of a significant increase in pSmad-1/5/8 and inhibitory Smad6, and decrease in αSMA suggest that anti-fibrotic effects of BMP7 in the cornea are mediated through the suppression of TGFβ-driven profibrotic Smad signaling by increasing the expression of inhibitory Smad6.

## Discussion

Conventional non-viral vectors have shown poor transfection efficiency and weak transgene delivery into corneal cells. According to many *in vitro* studies GNPs can serve as efficient gene delivery vectors [12]–[15]. However, few studies have tested their gene delivery potential using *in vivo* preclinical disease models. Herein, we demonstrate that GNPs can deliver therapeutically relevant levels of transgene into cornea *in vivo* without causing keratocyte cytotoxicity and inflammation. Our results show that PEI2-GNPs-mediated BMP7 gene delivery attenuates laser ablation-induced corneal fibrosis *in vivo* in a rabbit model.

BMP7 is a multifunctional cytokine that has a wide range of effects on cell growth, differentiation and apoptosis [21], [22], [43] and thus plays a pivotal role in the development of many organs including eyes during embryogenesis [28], [29]. However, BMP7 expression in most organs declines with age during adulthood and no BMP7 expression could be detected in the adult mouse cornea [32]. However, many non-ocular tissues that show low or no BMP7 expression have functional BMP7 signaling that can be activated by exogenous BMP7 administration [30], [44]. Therefore, we wanted to confirm that the observed antifibrotic effect due to BMP7 in the cornea is mediated through the activation of its receptor signaling. Indeed, the detection of phospho-Smad1/5/8 in the present study confirms the presence of a functional BMP signaling in the corneal fibroblasts. Activation of BMP7 signaling has been reported to oppose TGFβ biological activity [21], [22] and administration of recombinant BMP7 has been shown to reverse TGFβ hyperactivity driven fibrosis in organs such as the kidney and cardiac tissue [31], [45]. On the other hand, BMP7 was ineffective to treat skin and lung fibrosis suggesting that the antifibrotic effects of BMP7 may be organ dependent [46]. TGFβ levels in the tear fluid and corneal stroma are reported to substantially increase after laser surgery [47], [48]. Our group and others have shown that TGFβ is a significant player in the laser surgery induced fibrosis in the cornea [48], [49]. Based on its anti-TGFβ properties, we hypothesized that BMP7 gene therapy will attenuate corneal fibrosis. A significant decrease in corneal haze and myofibroblast formation in the GNP-BMP7 treated rabbits confirm our hypothesis that BMP7 gene therapy is an effective treatment for corneal fibrosis. We have previously demonstrated that therapeutic strategies that can intercept TGFβ before it activates its signaling are effective to treat corneal fibrosis [50], [51]. BMP7 is shown to inhibit TGFβ by blocking its signaling pathway. We and others have demonstrated that Smad7 over expressing human corneal fibroblasts (unpublished observation) and Smad6 over expressing mesangial cells [44] are resistant to the profibrotic effects of TGFβ, thus raising the possibility that BMP7 may inhibit TGFβ signaling by increasing the expression of the these anti-TGFβ Smads. This is supported by the significant increase in Smad6 expression in HCF after BMP7 gene therapy.

Depending upon dose and developmental stage, both pro and antiapototic effects of BMP7 have been reported for a variety of cell types including lens, vertebrae, and kidney [52]–[54]. In the present study, BMP7 gene therapy did not induce keratocyte apoptosis suggesting that BMP7 gene therapy is safe for the cornea. Additionally, BMP7 is reported to have an anti-inflammatory effect by reducing the expression of proinflammatory genes such as interleukin (IL) 6, IL1 and monocyte chemotactic protein-1 [55]. Epithelial scraping and laser ablation are documented to initiate a transient influx of inflammatory cells that are known to contribute to corneal scarring. Thus, it seems likely that besides BMP7’s anti-TGF effects, its anti-inflammatory properties may also contribute to its antifibrotic effects in the cornea. However, the inflammatory phase after laser ablation is transient and we did not detect any significant difference in the inflammatory cells in BMP7 treated corneas at 4 weeks.

In summary, this study demonstrates that PEI2-GNPs are an efficient vector for corneal gene therapy, and PEI2-GNPs mediated BMP7 gene delivery attenuates corneal fibrosis *in vivo* in rabbit eyes with minimal cytotoxicty or inflammatory response by counter balancing TGFβ-driven profibrotic Smad signaling. Furthermore, this study suggests that localized and targeted gene delivery approach could be used for studying functions of specific genes in corneal wound healing modulation *in vivo*.
